# Re-examining physician-scientist training through the prism of the discovery-invention cycle

**DOI:** 10.12688/f1000research.21448.1

**Published:** 2019-12-19

**Authors:** Gopal P. Sarma, Allan Levey, Victor Faundez

**Affiliations:** 1 Broad Institute of MIT and Harvard, Cambridge, MA, 02142, USA; 2 Analytic and Translational Genetics Unit, Massachusetts General Hospital, Boston, MA, 02114, USA; 3 Department of Neurology, Emory University School of Medicine, Atlanta, GA, 30322, USA; 4 Department of Cell Biology, Emory University, Atlanta, GA, 30322, USA

**Keywords:** metascience, translational research, biomedical policy, innovation, medical education, data science, MD/PhD, bench-to-bedside, physician-scientist, clinical research

## Abstract

The training of physician-scientists lies at the heart of future medical research. In this commentary, we apply Narayanamurti and Odumosu’s framework of the “discovery-invention cycle” to analyze the structure and outcomes of the integrated MD/PhD program. We argue that the linear model of “bench-to-bedside” research, which is also reflected in the present training of MD/PhDs, merits continual re-evaluation to capitalize on the richness of opportunities arising in clinical medicine. In addition to measuring objective career outcomes, as existing research has done, we suggest that detailed characterization of researchers’ efforts using both qualitative and quantitative techniques is necessary to understand if dual-degree training is being utilized. As an example, we propose that the application of machine learning and data science to corpora of biomedical literature and anonymized clinical data might allow us to see if there are objective “signatures” of research uniquely enabled by MD/PhD training. We close by proposing several hypotheses for shaping physician-scientist training, the relative merits of which could be assessed using the techniques proposed above. Our overarching message is the importance of deeply understanding individual career trajectories as well as characterizing organizational details and cultural nuances to drive new policy which shapes the future of the physician-scientist workforce.

## Introduction

The origin of the modern physician-scientist lies in the explosive growth of knowledge following the Second World War. Although a recognizable vision for the dual-degree training of clinical investigators was articulated as early as the second decade of the 20th century, it was the launching of the National Institutes of Health (NIH)-backed Medical Scientist Training Program ( MSTP) in 1964 in the US that cemented the MD/PhD into the framework of modern medical research ( Harding *et al.,* 2017; Meltzer, 1909).

In the half-century since then, growth of the physician-scientist pipeline in the United States has expanded to nearly 120 schools, with over a third funded through the NIH ( Akabas *et al.,* 2018; Jeffe *et al.*, 2014). Paralleling this growth has been a steady stream of meta-scientific research aimed at analyzing the career trajectories of dual degree graduates. With titles such as “The MD/PhD Researcher: What Species of Investigator,” “Transforming Science Into Medicine: How Clinician–Scientists Can Build Bridges Across Research’s ‘Valley of Death,’” and “Challenges and opportunities for reinvigorating the physician-scientist pipeline,” the fundamental aim of this type of analysis is to use systematic, evidence-based investigation into the frontiers of medical research to inform actionable policy ( Andreassen & Christensen, 2018; Daye *et al.,* 2015; DeLuca *et al.*, 2016; O’Mara *et al.*, 2015; Roberts *et al.,* 2012; Rosenberg, 2008; Sklar, 2017; Sutton & Killian, 1996; Thiele, 2018; Weggemans *et al.,* 2019).

In this commentary, we contribute to this knowledge-base by applying the framework of “discovery-invention cycles,” as proposed by Venkatesh Narayanamurti and Toluwalogo Odumosu, to the training of physician-scientists ( Narayanamurti & Odumosu, 2016). Their framework can be seen both as a theory of scientific progress as well as a theory of innovation—we will draw on both perspectives in this article. We begin by summarizing their argument for the inadequacy of the linear model of research and for dismantling the artificial distinctions between “basic” and “applied” research, which we equate with notions of “bench” and “bedside” research in the medical setting. We argue that the “bench-to-bedside” paradigm of clinical-translational research is a manifestation of the linear model, which informs the structure of the integrated MD/PhD program. We propose that re-framing clinical-translational research in the language of discovery-invention cycles allows us to better align the training of physician-scientists with 21 ^st^-century needs and opportunities.

Next, we examine the obstacles to accurately measuring outcomes in dual-degree programs. We argue that objective measures related to job description, such as employment at an academic center, percentage of time spent conducting research, and field of research, give a limited window into whether the unique training of dual-degree graduates is being utilized. As a complementary research paradigm, we propose that more in depth, anthropological research is necessary to understand the specific ways in which individuals are or are not taking advantage of their clinical and scientific background. As a set of tools to aid qualitative investigation, we propose that machine learning and data science of large biomedical corpora along with anonymized clinical data may allow us to better understand if there are objective “signatures” of MD/PhD training.

Finally, we examine several pathways for re-thinking the training of physician-scientists: shifting the start of the PhD portion of MD/PhD programs to after the required clinical rotations; expanding the pipeline of residency/fellowship-based PhD programs; recruiting PhDs, other advanced degree holders, and professionals from relevant disciplines into the medical pipeline; and elevating the MD as a terminal clinical degree for a subset of individuals interested in non-clinical careers. Although most of these strategies are employed to varying degrees in existing programs, there is little objective basis on which to decide the relative proportion of each type of training in a national funding roadmap. Our proposals for collecting richer data will play a critical role in allowing policy makers in the biomedical sciences to better understand the consequences of different training paradigms. Taken together, the framing of the discovery-invention cycle, anthropological investigation, and the tools of modern data science will allow the medical establishment to navigate rapidly evolving intellectual frontiers at the interface of medicine and other disciplines.

Although much of our discussion is US-centric, our analysis is relevant for other countries as well, as the American model has largely been replicated elsewhere. Moreover, dual-degree physician-scientist training appears to play a significantly smaller role in the landscape of medical research outside of the US ( Alamri, 2016). Consider, for example, that in the UK, the first integrated MB/PhD program was introduced at the University of Cambridge in 1989. In Australia, the first combined MBBS ^1^/PhD was introduced at the University of Sydney in 1998, but was subsequently scaled back significantly in 2014. In Africa, the University of Cape Town recently created a program in which students apply for additional research training in a stepwise fashion extending from a bachelors in medicine, to a masters, and finally to a PhD. This cohort began its training in 2011 and career outcomes are not yet available ( Alamri, 2016; Kandiah, 2013; Katz *et al.*, 2014). For countries and regions with fledgling efforts in physician-scientist training, we believe that our analysis will prove to be useful in establishing a vision and long-term roadmap for creating sustained innovation in the biomedical workforce.

## The discovery-invention cycle

*It is apparent then that the natural sciences and engineering have long been interlinked and that practical pursuits have never been far from the acquisition of new knowledge. However, in contemporary US science and technology policy and in the governance of many of our mission-oriented research agencies, this simple idea has been forgotten and silos and boundaries have built-up, impeding the progress of knowledge and inventions. The status quo is unsustainable and unacceptable.*
  - Venkatesh Narayanamurti and Toluwalogo Odumosu in *Cycles of Invention and Discovery: Rethinking the Endless Frontier* ( Narayanamurti & Odumosu, 2016)

Narayanamurti and Odumosu’s basic tenet is a simple one. They observe that advances that are traditionally considered the domain of “fundamental” research are often richly intertwined with more “applied” or “development” related work. This observation has deep, practical implications for science innovation, training, and policy. Because of the assumption that fundamental research has a linear relationship with applied research, policymakers at universities, funding agencies, or industrial research labs assume that increasing the pipeline of fundamental research will necessarily increase practical output at the other end. However, the reality is far more complex. The road from fundamental insights to practical technologies or other applications is strewn with many more obstacles and opportunities than this simple picture allows for. Consequently, policies aimed at increasing innovation by attempting to engineer aspects of this linear pipeline may be less than successful. In particular, thinking in terms of the linear model can result in institutions that fail to adequately identify and/or resolve bottlenecks to science innovation and training.

We will return to the question of physician-scientist training in the subsequent section and argue that the bench-to-bedside paradigm is a manifestation of a linear model in need of revision. Before turning our attention to medicine, however, we first examine 12 Nobel Prizes in telecommunications technology and nuclear magnetic resonance (NMR) that Narayanamurti and Odumosu use to illustrate the richly interdependent and non-linear nature of basic and applied research ( Narayanamurti & Odumosu, 2016). There are several points worth understanding in taking a bird’s eye view of this Nobel Prize winning work.

**Figure 1.**
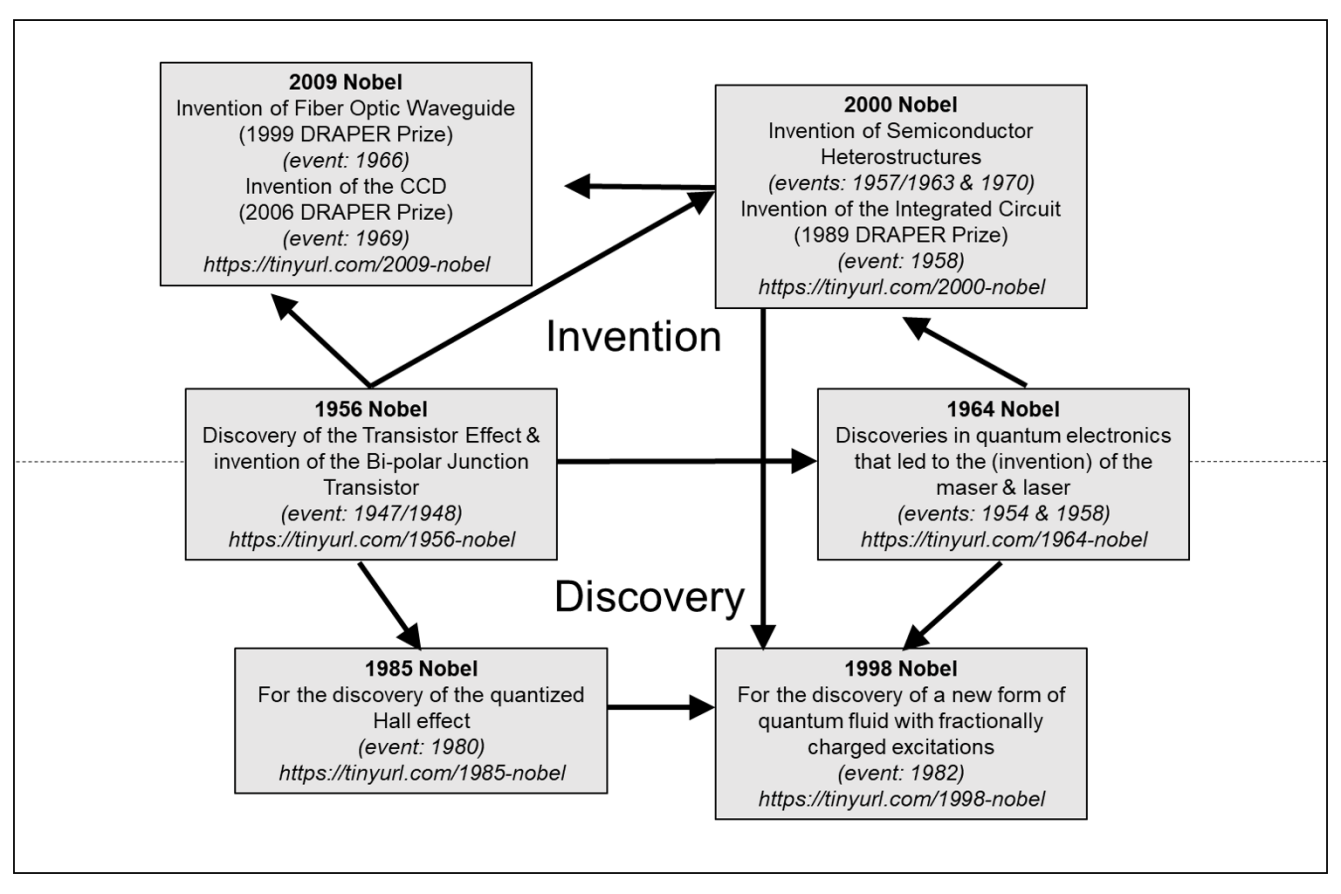
The discovery-invention cycle in information and communications technology. Arrows indicate conceptual dependencies among the various inventions and discoveries. Figure has been adapted with permission from ( Narayanamurti *et al.*, 2013).

The first is the complex interplay between advances firmly on the discovery side and those on the invention side. Most illustrative is the 1956 Nobel Prize in Physics, awarded to John Bardeen, William Shockley, and Walter Brittain for the discovery of the transistor effect ( Riordan *et al.*, 1999). In Figure 1, this award is firmly situated at the boundary of the discovery and invention territories. The reason is that not only did the transistor effect represent a fundamental advance in understanding interactions between electromagnetic fields and metals, but in addition, demonstrating the effect required *building* the bipolar-contact transistor. Indeed, eminent theorist John Bardeen spent countless hours alongside his experimental colleagues at Bell Laboratories during the development of this groundbreaking work. Likewise, the development of nuclear magnetic resonance (NMR), a discovery which ultimately came to transform medicine, required parallel insights into engineering technology, which would in turn allow further theoretical developments in quantum physics and quantum chemistry ( Boesch, 2004; Tabet & Rebuffat, 2003). The development of the molecular ray method and the discovery of the proton magnetic moment played an analogous role to NMR as the transistor did for entire branches of solid state physics, and consequently, are situated firmly on the boundary between discovery and invention ( Stern, 1946; see Narayanamurti & Odumosu, 2016 for an analogous diagram of interconnected Nobel Prizes leading to NMR).

The second point that Narayanamurti and Odumosu conclude from these historical developments is that building an institutional culture that allows such interdependent connections to be formed requires administrators to maintain both an overarching thematic focus along with a flexible and open-minded view towards the necessary diversity of prior training and experiences on a team. As the above examples are meant to illustrate, the series of steps that were required for this ground-breaking research to be conducted often took a meandering path spanning theoretical work, laboratory experiments, and practical industrial development. A rigid environment that prioritized either research publications, patents, or commercial developments to the exclusion of the others would have failed to deliver on the true potential of this work.

We summarize the basic takeaways from the discovery-invention cycle paradigm here:

1. All aspects of knowledge production should be intrinsically valued. Realizing the social impact of any advance requires many intricate steps, which can intertwine theoretical research, fundamental science, technological or product development, as well as managerial and organizational insights. A key element is the convergence of diverse intellectual assets.
2. The distinction between basic and applied research (or bench and bedside, in the case of medicine)—and the greater prestige that modern society affords either basic or applied research, depending on the context—can be detrimental to efficient resource allocation across the intellectual landscape.
3. Creating an institutional culture that maximizes the potential for innovative impact requires breaking down artificial boundaries and enabling dialogue not just across disciplines, but across the different dimensions of the discovery-invention cycle.

## Measuring the impact of dual-degree training

*Despite the remarkable success of the biotechnology sector and the embrace of its approaches by the pharmaceutical industry, a functioning LSM [life sciences and medicine] continuum linking fundamental discovery and the care of patients remains, for the most part, a goal rather than a reality.*
  - ARISE2 Committee ( American Academy of Arts and Sciences, 2013)

### The linear assumptions of “bench-to-bedside” research

The discovery-invention cycle can be considered both a theory of scientific progress as well as a theory of innovation. In applying this framework to medicine, we take the latter perspective and consider that at its core, innovation consists of novel amalgams of existing concepts, skills, methodologies, and resources, which in turn give rise to fundamentally new ones ( Baregheh *et al.,* 2009; Fagerberg *et al.,* 2005). Theoretically, the greater the number, diversity, and depth of the clinical and scientific training of individuals, the greater the possibilities for novel combinations of concepts, skills, methodologies, and resources. We see the original vision of the MD/PhD as being in this spirit—to increase innovative outcomes at the interface between basic science and clinical research.

Physician-scientists are meant to play the unique role of bridge builders in the vast landscape of the basic and clinical sciences ( American Academy of Arts and Sciences, 2013; Zemlo *et al.,* 2000). What is the nature of these bridges and how should we train future leaders to help construct these key edifices? The notions of “bench to bedside” and “bedside to bench” hide the complex realities which underlie both of these research and development pathways. A ‘pure’ example of formulating testable hypotheses from clinical observations is John Snow’s discovery of the causes of cholera outbreaks in 19 ^th^ century London, even before the advent of the germ theory ( Johnson, 2006). Likewise, it is expected that physician-scientists enable the subsequent steps of translational research, which will give rise to novel therapies from bench research.

Unfortunately, both sides of this process invariably involve complex detours that hamper the attempts of the well-trained physician-scientist. One example of the tortuous nature of both the “bench to bedside” and “bedside to bench” trajectories is manifest in psychiatric genetics. Schizophrenia and mood disorders are characteristically polygenic, thus preventing the formulation of biological pathogenesis hypotheses from classical Mendelian genetic approaches ( International Schizophrenia Consortium *et al.,* 2009). It was the advent of powerful chemical DNA synthesis tools, DNA hybridization on chips, DNA sequencing technologies, robotics, novel statistical tools, and the computer power to analyze and store genomic and clinical data from thousands of patients and controls that has opened the door to formulate biological hypotheses of the underlying disease mechanisms ( Geschwind & Flint, 2015; Sullivan *et al*., 2012). While it is not our aim to undertake an analysis as thorough as Narayanamurti and Odumosu, there is no shortage of clinical breakthroughs whose trajectory was as meandering and boundary crossing as the advances in solid state physics and NMR summarized above. Consider the example of statins, drugs that are widely used to treat hypercholesterolemia. Their discovery and clinical applicability needed the convergence of knowledge about natural product chemistry, cholesterol metabolism, and the genetics of hypercholesterolemia disorders, to mention just a few ( Stossel, 2008). In both of these cases, schizophrenia and statins, fundamental science, technological / product development, and managerial / organizational insights have been key enablers of research progress.

On the one hand, many will point out that these observations are hardly surprising. To some degree, the diversity of efforts required to actualize a given medical therapy—basic or applied, academic or industrial—is a well understood phenomenon. On the other hand, if so, is this reality reflected in how we train our physician-scientists? And what of the expected career trajectories of dual-degree graduates? Does the ideal physician-scientist career path optimally take advantage of the unique nature of their training? What policy changes can be implemented to fully realize the intellectual potential of our medical establishment and our arsenal of dual-degree graduates?

### The need for qualitative analysis

As we discussed in the introduction, there is a sizeable body of metascientific research analyzing the impact of MD/PhD training. Most recently, in the United States, the Association of American Medical Colleges conducted a thorough and thoughtful investigation into nearly 50 years’ worth of career outcomes of dual-degree graduates ( Akabas *et al.*, 2018). To assess the institution-wide impact, they examine a wide variety of measures including employment at an academic center, presence in major grant databases, and percentage of protected research time, among others. The AAMC study is an impressive first-pass analysis characterizing the landscape of dual-degree career outcomes. Given the complex, multi-faceted nature of physician-scientist training and practice, their report paves the way for subsequent investigations that can build on this foundation.

One of the conclusions of the AAMC study is that many questions remain in understanding the nature of the research landscape of physician-scientists. As an example, although the authors went into the study expecting a bimodal distribution of research time (i.e. with most being primarily clinicians or pure researchers), they discovered that there is in fact a continuum of research effort. Beyond a high-level description of their research activities, a major open question remains as to how researchers are taking advantage of their training apart from maintaining parallel job descriptions in varying proportions.

Our primary takeaway from applying the lessons of the discovery-invention cycle to medical research is that there is a significant need for in-depth qualitative analysis to better characterize whether dual-degree training is truly being utilized. Even if we can establish beyond the shadow of a doubt that MD/PhDs are deeply involved in both clinical and research activities, there is still the lingering question of whether or not they are leveraging their unique and extensive training. If we were to eliminate the dual-degree trajectory entirely, would the corresponding research output vanish, or would it be accomplished in different ways by pure MD and PhD researchers? Or is it that the true contribution of MD/PhDs is to facilitate dialogue between researchers and clinicians through higher-level administrative and organizational activities? The AAMC study is an impressive cross-sectional examination of the entire cohort of MD/PhD trainees giving policymakers high-level structural insights and subsequent analyses can be more targeted and examine smaller groups of individuals in significantly greater depth. Examining the roles, activities, and intellectual philosophy of physician-scientists even from a single academic organization (school, department, or division) would give significant insight into the impact of the training.

### Open data, machine learning, and data science

The application of data science to public corpora of scholarly publishing is allowing researchers to investigate novel hypotheses about the scientific process itself. These new research trends are being embraced in the life sciences (see, for example, the Meta-Research collection in the journal *eLife*). We can now analyze complex datasets to study the evolution of scientific language since the 19th century or the frequency of medical reversals in the literature on clinical trials, and use the results to inform policy decisions ( Herrera-Perez *et al.,* 2019; Plavén-Sigray *et al.,* 2017).

Likewise, we argue that modern computational techniques may have a unique role in augmenting the qualitative, anthropological analyses we have argued for in this piece. Indeed, quantitative techniques have a long history in the study of innovation ( Hall & Jaffe, 2012; National Academies of Sciences, Engineering, and Medicine, Division of Behavioral and Social Sciences and Education, and Committee on National Statistics, 2017). Imagine that we have a fully-digitized corpus of biomedical literature that can be used by data scientists. Such a resource would consist of aggregated articles from biomedical journals along with the tools for conducting large scale text searches, visualization, and the training of machine learning models. Is it possible that lying hidden in such datasets are signatures of research uniquely enabled by MD/PhD training?

One way to approach this question is from a purely exploratory standpoint. What are the patterns that emerge when looking at the research output of MD/PhD investigators? Are there differences in the distributions of journals that they publish in relative to pure MDs or PhDs? What about the distribution of topics? What about correlating trends in academic publishing from MD/PhDs with other sources, such as biomedical news or patent databases?

Another way to approach this question is the paradigm of classification ( Bishop, 2006). Suppose for every research publication in our dataset, we extract the educational qualifications of the authors (since many medical journals require this, we might even restrict to those papers that list the qualifications of authors). We can then use supervised learning to create models that predict whether a dual-degree graduate co-authored the research study. To simplify the analysis, we could focus on the first author, senior author, or perhaps train a model to predict how many MD/PhD degree holders were involved with the study. How accurate might such a model be? If it turns out that no such model can be trained to perform better than chance, does that imply that the research output of MD/PhDs could just as well have come from pure MDs or PhDs? On the other hand, if it turns out that a model can be trained to achieve high classification accuracy, does it imply the converse? Or might there be some latent structural explanation subtly biasing the model? These are all tantalizing questions that would require deeper investigation and which would add significant depth and new dimensions to the types of questions identified above.

Even more powerful would be to combine corpora of biomedical literature with anonymized medical records and even patent databases. For example, are there differences in documentation patterns between pure MDs versus MD/PhDs? What about in the standard of care? One would hope that there are no differences in routine cases, but what about in difficult situations where there is no standard of care? Are MD/PhDs able to leverage their knowledge and experience with the scientific literature to impact patient care? Analogous questions might be asked using combinations of the biomedical literature with patent databases to understand the role of MD/PhDs in the process of translating discovery to invention.

We make this proposal with some amount of trepidation and emphasize that the types of analysis proposed here need to be anchored to solid, anthropological investigation. Without a deep understanding of the context and culture of MD/PhD training, purely data-driven investigations might easily be misinterpreted and give credence to erroneous policy decisions.

## Training proposals

*Physician-scientists are trained to ask clinically relevant questions in a health research environment that lead to the development of research projects linking basic and clinical sciences. Physician-scientists are also a vital force in transforming clinical observations into testable research hypotheses and translating research findings into medical advances.*
  - Tamara R. Zemlo, Howard H. Garrison, Nicola C. Partridge, and Timothy J. Ley in *“The Physician-Scientist: Career Issues and Challenges at the Year 2000”* ( Zemlo *et al.,* 2000)

We now turn to several proposals to align the training of physician-scientists with the insights of the discovery-invention cycle. As we have discussed in the previous section, understanding the impact of different training paradigms is itself a complex question with a many-year time horizon from intervention to effect. Therefore, we present these proposals partly as hypotheses or topics for discussion that merit greater investigation using some of the techniques identified above. Several of the proposals we discuss have been implemented to varying degrees at existing universities. However, with the integrated MD/PhD emerging as the dominant form of physician-scientist training, it remains an open question what the relative proportions should be of the different pathways. Again, the techniques proposed above may prove useful in answering this question.

### For traditional MD and MD/PhD pipelines

*For existing integrated MD/PhD programs, shifting the start of the PhD until after a full year of clinical rotations. For the traditional MD to residency track, expanding the pool of residency/fellowship-based PhD programs. In both cases, encouraging the PhD student to have both a true research mentor and a true physician-scientist mentor.*

One of the most significant drawbacks of the linear model of research and development is a failure to adequately identify bottlenecks in the convoluted path from discovery to invention. Unfortunately, the current model of integrated MD/PhD training solidifies this model by sequentially training students in the pre-clinical sciences, followed by basic research, followed by clinical rotations. In the vast majority of cases in the US, students begin their PhD after having completed the pre-clinical years and taken Step 1 of the United States Medical Licensing Examination (USMLE), but before their required third-year clerkships.

This setup results in students choosing their PhD topic having had a limited exposure to the actual practice of clinical medicine. Consequently, students are not adequately prepared to identify a research path that is informed by experience with medicine itself. Shifting the start of PhD training to after the completion of third-year clerkships would give students substantially better preparation to choose a PhD area that would best serve their vision of being a physician-scientist in the context of an area of clinical scholarship that they wish to embark upon.

At some schools that have moved to an accelerated format for the pre-clinical years, in which Step 1 of the USMLE is taken after only 1–1.5 years of coursework, MD/PhD students are beginning their PhD after all or some of the clinical clerkships. See, for example, the MSTP program at Duke University, which has a one-year pre-clinical curriculum, or Baylor University, which has a 1.5-year pre-clinical curriculum. We endorse this path and our recommendation stands for schools that have maintained the traditional two-year format for pre-clinical training. For schools that have adopted a 1.5-year pre-clinical curriculum, but where MD/PhD students begin their graduate studies immediately after Step 1 of the USMLE, we recommend completing six months of clinical clerkships along with their traditional MD classmates prior to beginning their PhD, and perhaps also including 1–2 months of elective rotations in the areas relevant to the research they expect to pursue. An additional benefit of this arrangement is that students would be able to maintain their clinical skills during their PhDs. For example, an afternoon in an outpatient clinic once or twice a month would allow them to remain in contact with the clinical world while in the depths of their PhD research. It would also provide a valuable point of structure and regularity in the otherwise open-ended (and often soul searching!) years of graduate training.

The motivation behind expanding the pool of residency/fellowship-based PhD programs is similar in spirit. By residency/fellowship-based PhD programs, we are conceiving of a curriculum in which the majority of a student’s research is done during dedicated years separate from clinical training. We are skeptical that quality research can be conducted entirely in parallel with the grueling demands of residency and fellowship. See the Stanford ARTS Program or the UCLA STAR Program for outstanding models of research curricula integrated with post-graduate medical training. In both cases, the motivation is to ensure that students have greater exposure to clinical medicine before beginning their graduate careers. Moreover, residency/fellowship-based PhD programs open the door to many more students whose interest in a research career took a longer time to develop than those who decided early in their college careers apply to an integrated MD/PhD program.

Residency/fellowship-based PhD programs also carry the benefit that students are more mature than had they began their PhDs soon after completing their undergraduate degree. Such students are likely to bring a more informed perspective to their graduate studies. Compared to typical first-year graduate students, they will already have substantial exposure to the scientific and clinical literature and will have been surrounded by research for many years, even if they have not yet been immersed in it. Moreover, because they are close to being fully-trained physicians, they can continue their clinical responsibilities during their PhD years in outpatient clinics or while moonlighting.

For residency/fellowship-based PhD programs, many will correctly point out that the elephant in the room is length of time it takes to train and the corresponding financial implications. Most residents interested in pursuing a PhD did not have the benefit of a subsidized MD/PhD program, and thus are saddled with significant loans from medical school. They are also at a time in life when they may have a new marriage, are planning a family, etc., with competing demands on time and other resources. It becomes very difficult for most individuals in this situation to turn down a starting job with a well-remunerated salary, and taking several more years to pursue the PhD. There will likely need to be innovative, but perhaps costly solutions to deal with these realities. However, we remain optimistic that such solutions are possible. Individuals frequently make career decisions that are far from optimal financially, but which give them a deep satisfaction in their life choices. Even a career in academic medicine, particularly in the setting of a dual-income household, is more than livable, and we suspect that a small, but sizeable student body would be enthusiastic to participate in such a set of programs.

In both integrated MD/PhD and residency/fellowship-based PhD settings, appropriate forms of mentorship would be necessary to have maximal impact. Specifically, it should be encouraged for students to have both a research mentor and a physician-scientist mentor.

### For non-traditional MD candidates

*Creating a pipeline in which PhDs in other subjects or professionals of any kind with advanced training are recruited to the medical pipeline. For non-traditional MD candidates, ensuring a culture of mentorship and freedom that will allow them to develop their vision for integrating their prior experiences into a medical career.*

One of the aims of viewing physician-scientist training from the lens of the discovery-invention cycle is to enable medical culture to be flexible in the face of a rapidly evolving landscape and emerging interfaces with other subjects. We believe that a sensible approach to creating such innovation and agility that would require minimal administrative overhead is to recruit PhDs as well as professionals from other disciplines to the standard medical pipeline. There is a long history of individuals who have made the decision to transition from a PhD to an MD. However, what we are advocating is a more ambitious and targeted recruiting culture within the medical establishment itself. The significant challenge, therefore, is how to discern those who are committed to a career as a physician-scientist, which is the minority, from those who have found they no longer see themselves pursuing a career in research and want a new direction in life.

Actively identifying and recruiting highly trained professionals (PhDs or otherwise) has the advantage that individuals bring a mature worldview to medical school and set of novel skills that can inform their clinical training and research focus. A small number of schools have created accelerated MD programs for biological science PhDs. See for example, the 3-Year PhD-to-MD-Program at Columbia University. Moreover, in situations where there is an immediate need for insights from a field outside of medicine, trained professionals can begin to make small contributions from the very beginning and are no more than four years away from becoming resident physicians. On the other hand, recruits to the standard MD/PhD pipeline are many years away from being able to contribute their knowledge base to medicine, even if their PhD topic has been strategically chosen to address a current need. Certainly, some creativity will be necessary to execute such a recruitment process, given the personality, skill set, and passion required for success in clinical medicine. However, considering that the demands and rewards of medicine are well understood by the educated public, and given the long history of individuals in medicine who have gone through significant career changes, we are confident that such a targeted recruitment process is achievable by sufficiently motivated medical school admissions committees.

As one contemporary example, we mention the intensely controversial discussions surrounding electronic health records (EHRs). Many believe that EHRs have negatively impacted the job satisfaction of healthcare workers as well as patient satisfaction. Indeed, research articles have specifically examined the role that EHRs play in increasing burnout among physicians ( Arndt *et al.,* 2017; DiAngi *et al.*, 2016; Shanafelt *et al.,* 2016). However, among the many voices in this discussion, we observe that one voice is notably absent: that of professional software engineers and user interface designers who could lend insights into why the situation in medicine has had such a disappointing outcome when we are surrounded by high-quality software in other arenas. Recruiting professional software engineers to the medical pipeline would allow these highly skilled individuals to contribute their insights to medical culture and establish the foundations for future advances at the intersection of software, medicine, and artificial intelligence.

The importance of mentorship in this process cannot be overstated. Unlike mathematics or theoretical physics, where one can be a prodigious scientist at a young age, the human and institutional complexity of medicine means that years of experience are typically required before an individual can speak with authority and generate mature insights. For those who are entering medical training after having established themselves in another field, being mentored by someone experienced in medicine can play a significant role in finding avenues to allow their existing knowledge base to become intertwined with medicine.

There are several exciting developments in science publishing that can be powerful enablers of this process. The use of “pre-prints” as part of the life cycle of idea generation, a practice that has been commonplace in some subjects for nearly 30 years, and which is now becoming more common practice in biomedicine, opens the door for experienced medical students, such as those with PhDs in other areas, to write scholarly articles on topics of medical interest but from a novel and integrated perspective that become part of the citable scientific literature. Likewise, the emergence of “open science,” and large-scale, distributed scientific collaborations may be particularly valuable ways for trained professionals to identify topics and projects of relevance to medicine that may not yet exist at their home institution ( Nielsen, 2011; Sarma & Faundez, 2017).

### For both traditional and non-traditional candidates

*Encouraging a culture that values the MD as a terminal degree for individuals interested in non-clinical career paths such as medical data science, product development, consulting, science journalism, program management, venture capital or other positions that would advance the discovery-invention cycle across the life sciences.*

An implicit assumption in analyses of the integrated MD/PhD program is that ideal outcomes involve individuals who maintain dual clinical and research responsibilities. However, this assumption rests on a linear view of bench-to-bedside research in which the role of the physician-scientist is to make testable hypotheses at the bench based on observations made at the bedside. If the reality of this pathway is far more complex, then perhaps it is not simply the specifics of training that should be reconsidered, but also the ideal career trajectory that individuals aspire to. Indeed, is it possible that success stories of dual-degree training may involve careers with neither research nor clinical responsibilities?

The dramatic growth of knowledge across the entire spectrum of the life sciences and the critical role played by novel technologies suggests that there are many important roles outside of academia that could be played by traditional dual-degree graduates as well as the non-traditional variants we have suggested above. Taking advantage of their strong skills as scientific generalists, they would play critical roles in overseeing product development at startups, managing portfolios of life science startups at venture capital firms, or leading data science efforts at health systems. The role of the physician-scientist training in these contexts is not necessarily to directly enable linking fundamental research in biology to patient care. Rather, it is to help manage the burgeoning institutional complexity of the life sciences by bringing significant intellectual depth that encompasses diverse facets of a biomedical education.

There are many individuals who have created non-traditional career paths building on MD or MD/PhD training without pursuing additional post-graduate experiences ( Moawad, 2013). However, there may be significant value in a more systematic approach to training and encouraging such individuals. One possibility might consist of a 2–3 year program that encompasses a medical internship along with rotations with companies spanning different verticals in the life sciences. Such a post-graduate education would allow individuals to continue to strengthen their core clinical competencies while also giving them a survey of the many dimensions required to realize the bench-to-bedside vision.

## Discussion

The creation of the integrated MD/PhD program showed tremendous vision and foresight among policymakers in the middle of the 20 ^th^ century. Confronting an exploding knowledge-base and a shifting organizational landscape due to the professionalization of science, medical and scientific leaders realized that capitalizing on the full capabilities of post-WWII institutions required training generalists who could navigate the complexities of both the worlds of basic science and clinical medicine. We are in a fortunate position, therefore, to look back on over a half a century’s worth of hard data and accumulated cultural wisdom about the successes and impact of these programs. The paradigm of the discovery-invention cycle, which itself is a data-driven framework arising from decades of experience with academic and industrial science, is an ideal lens with which to re-examine the fundamental basis of physician-scientist training.

As the authors of the ARISE2 report quoted above argue, a continuum linking fundamental discovery to patient care remains a vision rather than a reality ( American Academy of Arts and Sciences, 2013). Our thesis is that significantly richer analysis of the specific activities of physician-scientists is necessary to understand whether dual-degree training has contributed to linking these two domains. In addition to qualitative analysis, we argued for assembling biomedical datasets of research output and clinical data, which would allow policy researchers to investigate if there are objective “signatures” of physician-scientist training. We hope that the considerations we have identified here are, at the very least, compelling starting points for widespread discussion of this important topic, and we are eager to collaborate with others to refine and implement these ideas so as to positively impact the future of physician-scientist training.

## Data availability

No data are associated with this article.

## Footnotes

^1^MBBS stands for Bachelor of Medicine / Bachelor of Surgery in countries where medical training is awarded an undergraduate degree.

## References

[ref-1] Akabas MH Tartakovsky I Brass LF: The National MD--PhD Program Outcomes Study. American Association of Medical Colleges Reports. 2018.

[ref-2] Alamri Y: The Combined Medical/PhD Degree: A Global Survey of Physician-Scientist Training Programmes. *Clin Med (Lond).* 2016;16(3):215–18. 10.7861/clinmedicine.16-3-215 27251908 PMC5922697

[ref-3] American Academy of Arts and Sciences: ARISE II: Unleashing America’s Research & Innovation Enterprise.2013 Reference Source

[ref-4] Andreassen P Christensen MK: Science in the Clinic: A Qualitative Study of the Positioning of MD-PhDs in the Everyday Clinical Setting. *BMC Med Educ.* 2018;18(1):115. 10.1186/s12909-018-1222-2 29801484 PMC5970524

[ref-5] Arndt BG Beasley JW Watkinson MD: Tethered to the EHR: Primary Care Physician Workload Assessment Using EHR Event Log Data and Time-Motion Observations. *Ann Fam Med.* 2017;15(5):419–26. 10.1370/afm.2121 28893811 PMC5593724

[ref-6] Baregheh A Rowley J Sambrook S: Towards a Multidisciplinary Definition of Innovation. *Management Decision.* 2009;47(8):1323–39. 10.1108/00251740910984578

[ref-7] Bishop CM: Pattern Recognition and Machine Learning. Springer. 2006 Reference Source

[ref-8] Boesch C: Nobel Prizes for Nuclear Magnetic Resonance: 2003 and Historical Perspectives. *J Magn Reson Imaging.* 2004;20(2):177–79. 10.1002/jmri.20120 15269938

[ref-9] Daye D Patel CB Ahn J: Challenges and Opportunities for Reinvigorating the Physician-Scientist Pipeline. *J Clin Invest.* 2015;125(3):883–87. 10.1172/JCI80933 25689260 PMC4362227

[ref-10] DeLuca GC Ovseiko PV Buchan AM: Personalized Medical Education: Reappraising Clinician-Scientist Training. *Sci Transl Med.* 2016;8(321):321fs2. 10.1126/scitranslmed.aad0689 26764155

[ref-11] DiAngi YT Longhurst CA Payne TH: Taming the EHR (Electronic Health Record) - There Is Hope. *J Fam Med.* 2016;3(6):pii: 1072. 27830215 PMC5098336

[ref-12] Fagerberg J, Professor of Business and Public Policy in the Walter a Haas School of Business David C Mowery, David C. Mowery, *et al.*: The Oxford Handbook of Innovation.Oxford University Press.2005 Reference Source

[ref-13] Geschwind DH Flint J: Genetics and Genomics of Psychiatric Disease. *Science.* 2015;349(6255):1489–94. 10.1126/science.aaa8954 26404826 PMC4694563

[ref-14] Hall BH Jaffe AB: Measuring Science, Technology, and Innovation: A Review. *Report Prepared for the Panel on Developing Science, Technology, and Innovation Indicators for the Future.* 2012 Reference Source

[ref-15] Harding CV Akabas MH Andersen OS: History and Outcomes of 50 Years of Physician-Scientist Training in Medical Scientist Training Programs. *Acad Med.* 2017;92(10):1390–1398. 10.1097/ACM.0000000000001779 28658019 PMC5617793

[ref-16] Herrera-Perez D Haslam A Crain T: A comprehensive review of randomized clinical trials in three medical journals reveals 396 medical reversals *eLife.* 2019;8:pii: e45183. 10.7554/eLife.45183 31182188 PMC6559784

[ref-17] International Schizophrenia Consortium, Purcell SM Wray NR: Common Polygenic Variation Contributes to Risk of Schizophrenia and Bipolar Disorder. *Nature.* 2009;460(7256):748–52. 10.1038/nature08185 19571811 PMC3912837

[ref-18] Jeffe DB Andriole DA Wathington HD: The Emerging Physician-Scientist Workforce: Demographic, Experiential, and Attitudinal Predictors of MD-PhD Program Enrollment. *Acad Med.* 2014;89(10):1398–1407. 10.1097/ACM.0000000000000400 25006709 PMC4175019

[ref-19] Johnson S: The Ghost Map: The Story of London’s Most Terrifying Epidemic and How It Changed Science, Cities, and the Modern World. Penguin. 2006 Reference Source

[ref-20] Kandiah DA: Sustainability of MBPhD Programmes. *Clin Med (Lond).* 2013;13(2):214. 10.7861/clinmedicine.13-2-214 23681880 PMC4952648

[ref-21] Katz AA Futter M Mayosi BM: The intercalated BSc (Med) Honours/MB ChB and integrated MB ChB/PhD tracks at the University of Cape Town: models for a national medical student research training programme. *S Afr Med J.* 2014;104(2):111–3. 24893538 10.7196/samj.7639

[ref-22] Meltzer SJ: The Science of Clinical Medicine: What It Ought to Be and the Men to Uphold It. *JAMA.* 1909;53(7):508–12. 10.1001/jama.1909.92550070001001b

[ref-23] Moawad H: Careers Beyond Clinical Medicine. OUP USA.2013 Reference Source

[ref-24] Narayanamurti V Odumosu T Vinsel L: RIP: The basic/applied research dichotomy.Issues Sci Technol.2013;29(2):31–36. Reference Source

[ref-25] Narayanamurti V Odumosu T: Cycles of Invention and Discovery.2016 10.4159/9780674974135

[ref-26] National Academies of Sciences, Engineering, and Medicine, Division of Behavioral and Social Sciences and Education, and Committee on National Statistics: Advancing Concepts and Models for Measuring Innovation: Proceedings of a Workshop.National Academies Press.2017 10.17226/23640

[ref-27] Nielsen M: Reinventing Discovery: The New Era of Networked Science.Princeton University Press.2011 Reference Source

[ref-28] O'Mara RJ Hsu SI Wilson DR: Should MD-PhD programs encourage graduate training in disciplines beyond conventional biomedical or clinical sciences? *Acad Med.* 2015;90(2):161–64. 10.1097/ACM.0000000000000540 25354071 PMC4310812

[ref-29] Plavén-Sigray P Matheson GJ Schiffler BC: The readability of scientific texts is decreasing over time. *eLife.* 2017;6:pii: e27725. 10.7554/eLife.27725 28873054 PMC5584989

[ref-30] Riordan M Hoddeson L Herring C: The Invention of the Transistor.In *More Things in Heaven and Earth.*Springer.1999;71(2):563–78. 10.1007/978-1-4612-1512-7_37

[ref-31] Roberts SF Fischhoff MA Sakowski SA: Perspective: Transforming science into medicine: how clinician-scientists can build bridges across research's "valley of death". *Acad Med.* 2012;87(3):266–70. 10.1097/ACM.0b013e3182446fa3 22373616

[ref-32] Rosenberg LE: MD/PhD programs--a call for an accounting *JAMA.* 2008;300(10):1208–9. 10.1001/jama.300.10.1208 18780852

[ref-33] Sarma GP Faundez V: Integrative biological simulation praxis: Considerations from physics, philosophy, and data/model curation practices. *Cell Logist.* 2017;7(4):e1392400. 10.1080/21592799.2017.1392400 29296511 PMC5739097

[ref-34] Shanafelt TD Dyrbye LN Sinsky C: Relationship Between Clerical Burden and Characteristics of the Electronic Environment With Physician Burnout and Professional Satisfaction. *Mayo Clin Proc.* 2016;91(7):836–48. 10.1016/j.mayocp.2016.05.007 27313121

[ref-35] Sklar DP: We Must Not Let Clinician-Scientists Become an Endangered Species. *Acad Med.* 2017;92(10):1359–1361. 10.1097/ACM.0000000000001870 28952984

[ref-36] Stern O: The Method of Molecular Rays. *Nobel Lecture.* 1946 Reference Source

[ref-37] Stossel TP: The discovery of statins. *Cell.* 2008;134(6):903–5. 10.1016/j.cell.2008.09.008 18805080

[ref-38] Sullivan PF Daly MJ O'Donovan M: Genetic architectures of psychiatric disorders: the emerging picture and its implications. *Nat Rev Genet.* 2012;13(8):537–51. 10.1038/nrg3240 22777127 PMC4110909

[ref-39] Sutton J Killian CD: The MD-PhD researcher: what species of investigator? *Acad Med.* 1996;71(5):454–59. 10.1097/00001888-199605000-00013 9114861

[ref-40] Tabet JC Rebuffat S: [Nobel Prize 2002 for chemistry: mass spectrometry and nuclear magnetic resonance]. *Med Sci (Paris).* 2003;19(8–9):865–72. 10.1051/medsci/20031989865 14593619

[ref-41] Thiele RH: Aligning Health Care and Academia: The Clinician Innovator. *Acad Med.* 2018;93(7):960–61. 10.1097/ACM.0000000000002248 29944541

[ref-42] Weggemans MM Friesen F Kluijtmans M: Critical Gaps in Understanding the Clinician-Scientist Workforce: Results of an International Expert Meeting. *Acad Med.* 2019;94(10):1448–54. 10.1097/ACM.0000000000002802 31135403

[ref-43] Zemlo TR Garrison HH Partridge NC: The physician-scientist: career issues and challenges at the year 2000. *FASEB J.* 2000;14(2):221–30. 10.1096/fasebj.14.2.221 10657979

