# Supplementary material for: Re-examining physician-scientist training through the prism of the discovery-invention cycle
Source: F1000Res. 2019 Dec 19;8:2123. [Version 1] doi: 10.12688/f1000research.21448.1 (PMC7014578; doi:10.12688/f1000research.21448.1)
Supplement: Peer review history

# Peer review: Reviewer response for version 1

**DOI:** 10.5256/f1000research.23626.r58687

**Published:** 2020-02-11

**Authors:** Bryan G. Yipp

**Affiliations:** 1 University Of Calgary, Calgary, Canada

This opinion paper highlights the continued challenges faced by practicing physician-scientists, MD/PhD trainees and training programs in general. Overall, the authors highlight ongoing questions including, what is the best way to train physician-scientists? What are the roles of physician-scientists? And what are the metrics of success for physician-scientists? 

 The first issues raised by the authors surround training programs and specifically about the formalized MD/PhD training programs in the United States. The introduction of the discovery-invention cycle is conceptually interesting; however, their discussion presumes that the formal combined MD/PhD program is the gold standard of physician scientist training. Indeed, this is an unproven idea that formalized MD/PhD programs are the optimal means to train physician-scientists. Since the formalization of the MD/PhD and MSTP in the US there has been steady increases in the number of trainees which has not resulted in the increase in working physician researchers. Therefore, it should be considered that the current training methods may not achieve the desired goals, if the desired goals can even be agreed upon. Interestingly, the authors use Nobel prize discoveries to illustrate their point about the discovery-invention cycle, but it should be noted that many Nobel Laureates have been MD trained without a PhD degree nor completing a formal MD/PhD combined program.

 The authors point out that a “rigid environment… would have failed to deliver on the true potential” in terms of the discovery-invention cycle; however, this same rigid environment may be having similar negative consequences to physician-scientist training. For example, there continues to be issues of diversity in the physician-scientist work force and within MD/PhD training programs. Data suggest that there are significant inequalities based on race, ethnic groups, socioeconomic status and sex. It remains uncertain if the rigid construct of MD/PhD training plays any role in these inequalities; however in our institution we have observed and published that training programs with flexible research pathways have resulted in increases in female medical doctors who work as researchers.

 Another rigid assumption made by the authors is that “physician-scientist are meant to play the unique role of bridge builders”. The assertion that physician researchers have a predestined role in biomedical research as “translationalists” is too constraining. First, physician-scientist have had very positive impacts on discovery and mechanistic research, again exemplified by MD Nobel Laureates. Indeed, my own postdoctoral supervisor Dr. Ralph Steinman was MD trained but went on to be awarded the 2011 Nobel prize in medicine for the discovery of dendritic cells. During a conversation about translational research, Dr. Steinman asked, “Why can’t physician-scientists just be allowed to discover things?” a question that helped shape my own research career. A second comment about this issue, is that translational research has benefited tremendously from PhD scientists without formal clinical training. Therefore, translation need not be owned by physician-scientists, nor should MD researchers be boxed-in to being translational scientists.

 The authors propose ideas to try to define what physician-scientists do and how to measure their unique impact on research and health. This includes defining metrics of success and outcomes of training programs. This is an interesting and very challenging area. Indeed, there is currently no consensus to the meaning of success. Many variables could be considered, including job position, publication numbers, grants, students, invited lectures and so on, but it is correctly pointed out that both qualitative and quantitative measures must be utilized. Despite this, there will likely never be agreement of the markers of success and this is not unique to physician-scientists but is also true for PhD scientists.

 The opinion article ends with some interesting ideas about future training pathways which are worth considering. Indeed, the concepts of scientist-physicians or those who study medicine but do not intend to practice, is an attractive alternate pathway.

I confirm that I have read this submission and believe that I have an appropriate level of expertise to confirm that it is of an acceptable scientific standard.

# Peer review: Reviewer response for version 1

**DOI:** 10.5256/f1000research.23626.r58411

**Published:** 2020-01-21

**Authors:** Paul A. Randazzo

**Affiliations:** 1 Section on Regulation of Ras Superfamily, Laboratory of Cellular and Molecular Biology, Center for Cancer Research, National Cancer Institute (NCI), National Institutes of Health (NIH), Bethesda, MD, USA

The paper opens two discussions. One is about the value of programs specifically designed to train physician/scientists, i.e. MD/PhD, with discussion of approaches to determine whether the programs achieve desired goals, while admitting that the goal of having a continuum of basic science and clinical research has not been achieved. 

 The second discussion is how to optimize training of physician/scientists, assuming, for instance, that MD/PhD programs have in place efforts to achieve the goal of the continuum of research as well as discussing other areas in which the dual degree may be valuable. Perhaps ironically, as they argue the case with the basic science and clinical training of MD/PhDs are not well integrated, neither are the two parts of this commentary.

 Questions that would be interesting to address include how the data they propose to collect determining whether the MD/PhD programs are achieving their goals would be used to inform redesign of the programs. They are assuming shortcomings in their proposed redesign of programs, but don't have data to support that, or at least it is not well articulated. Nevertheless, the discussion is worthwhile and the integration may not be possible. The other topic that might be included is how criteria for career advancement may influence achieving the goals of the programs. Financial issues are discussed, but the criteria used to advance and retain individuals with the training are not.

I confirm that I have read this submission and believe that I have an appropriate level of expertise to confirm that it is of an acceptable scientific standard.
